# Male breast cancer following complete remission of acute myeloid leukemia: A case report and literature review

**DOI:** 10.1016/j.ijscr.2025.111726

**Published:** 2025-07-23

**Authors:** Weiqiang Qiao, Peng Li, Chang Chang, Mengnan Fan, Miao Deng

**Affiliations:** a Department of Breast Surgery, The First Affiliated Hospital, and College of Clinical Medicine of Henan University of Science and Technology, Luoyang 471003, China; b Department of Pathology, The First Affiliated Hospital, and College of Clinical Medicine of Henan University of Science and Technology, Luoyang 471003, China; c Department of Medical Records and Statistics, The First Affiliated Hospital, and College of Clinical Medicine of Henan University of Science and Technology, Luoyang 471003, China

**Keywords:** Male breast cancer, Acute myeloid leukemia, Case report

## Abstract

**Introduction:**

Male breast cancer (MBC) is a rare clinical entity. The concurrent development of MBC and leukemia is particularly rare.

**Presentation of case:**

A 51-year-old male patient developed MBC following a 1-year complete remission of acute myeloid leukemia (AML). The patient underwent neoadjuvant chemotherapy, followed by surgical resection and adjuvant radiotherapy, and is currently receiving endocrine therapy with tamoxifen (10 mg twice daily, orally), with favorable outcomes observed during recent follow-up evaluations.

**Discussion:**

MBC is predominantly diagnosed as locally advanced disease. Reported cases include MBC following acute lymphoblastic leukemia (ALL) and therapy-related AML post-chemotherapy for female breast cancer, whereas our case exhibits novel distinctions compared to prior reports.

**Conclusion:**

This case report describes the first documented occurrence of MBC in a patient who achieved complete remission from AML, offering valuable insights for the clinical management of analogous cases in the future.

## Highlights

• This case report describes the first documented occurrence of male breast cancer (MBC) in a patient who achieved complete remission from acute myeloid leukemia (AML).
• The findings offer valuable insights for the clinical management of analogous cases in the future.
• We hypothesize that the series of chemotherapy regimens administered for this patient's AML treatment may have induced somatic gene mutations.

## 1 Introduction

Male breast cancer (MBC) represents a rare clinical entity, accounting for less than 1 % of all breast cancer cases [1]. The concurrent development of MBC and leukemia is particularly rare, with existing evidence predominantly documented in case reports [2,3]. Here, we report a case of MBC emerging post-complete remission in an acute myeloid leukemia (AML) patient. This case report has been reported in line with the SCARE checklist [4].

## 2 Case presentation

In February 2006, a 33-year-old male patient was diagnosed with AML in the Department of Oncology. Prior to the diagnosis of AML, the patient was in good health with no significant abnormalities detected during routine physical examinations. There was no notable family history of malignant tumors or hematologic disorders. The patient was diagnosed with AML with increased granules, classified as the M3 subtype (AML-M3a) according to the French-American-British (FAB) classification system. The patient achieved remission following induction chemotherapy with a arsenic trioxide and retinoid regimen. From 2006 to 2010, the patient underwent sequential chemotherapy regimens (daunorubicin and cytarabine, mitoxantrone and cytarabine, homoharringtonine and cytarabine, pirarubicin and cytarabine, etoposide and cytarabine), which resulted in sustained complete remission.

**Fig. 1:**
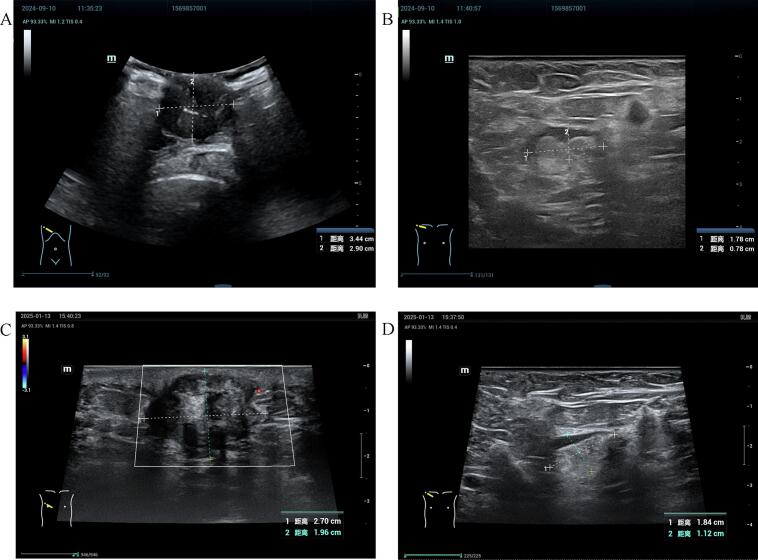
Ultrasonography of primary right breast mass (A) and right axillary lymph node (B) prior to neoadjuvant chemotherapy. Ultrasonography of the right breast mass (C) and right axillary lymph node (D) post-neoadjuvant chemotherapy.

**Fig. 2:**
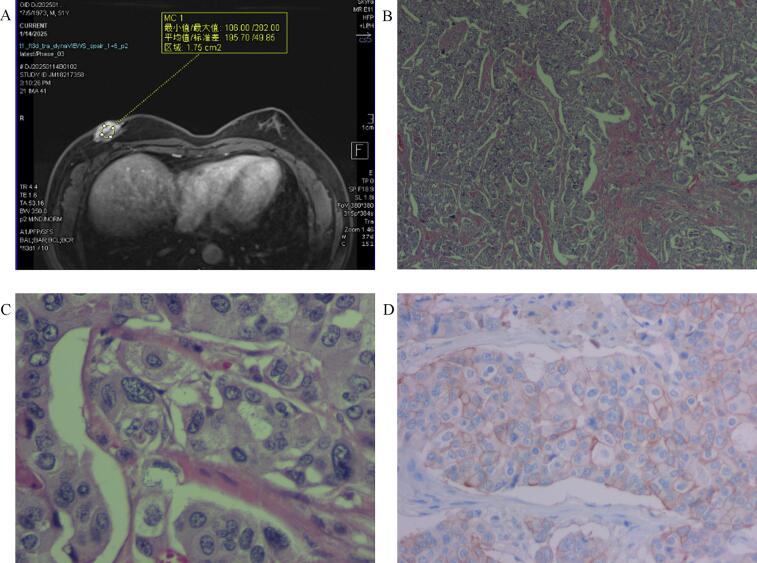
MRI image of the right breast (A). Clinicopathological examination of the resected breast tissue, immunohistochemical staining of the invasive ductal carcinoma; (H&E staining; magnification × 40) (B); (H&E staining; magnification × 400) (C); (HER-2 staining; magnification × 400) (D).

In September 2024, the patient was admitted to the Department of Oncology with a primary complaint of a right breast mass, which had been present for three years. Breast ultrasound identified a subareolar mass (34 mm maximal diameter) with ill-defined margins and heterogeneous echogenicity (Fig. 1A). An axillary lymph node (17 mm long-axis) showed cortical thickening and effaced hilum (Fig. 1B). Whole-body CT revealed no distant metastases. A pathological biopsy of the right breast mass revealed invasive ductal carcinoma of non-special type, with a histological grade of 2. Immunohistochemical analysis demonstrated estrogen receptor (ER) (90 % +), progesterone receptor (PR) (80 % +), human epidermal growth factor receptor 2 (HER-2) expression (2+, and fluorescence in situ hybridization (FISH) -), and Ki-67 (10 % +). Pre-chemotherapy clinical staging was cT4N2M0 (Stage IIIB). The patient underwent 6 cycles of neoadjuvant chemotherapy with a docetaxel and carboplatin regimen, achieving a clinical response assessed as partial remission. The patient was subsequently transferred to the Department of Breast Surgery for surgical intervention. Preoperative breast ultrasound identified the mass (27 mm maximal diameter) (Fig. 1C), and an axillary lymph node (18 mm long-axis) (Fig. 1D). Preoperative breast magnetic resonance imaging (MRI) identified a mass-like lesion adjacent to the nipple, measuring approximately 41 mm × 24 mm × 21 mm. The lesion displayed lobulated margins, and a washout pattern observed on the time-intensity curve (Fig. 2A). Post-chemotherapy clinical stage was confirmed as ycT4N2M0 (Stage IIIB). On January 17, 2025, the patient underwent a modified radical mastectomy for right breast cancer. Pathological examination confirmed invasive ductal carcinoma of the right breast, measuring 40 mm in diameter, with the Miller-Payne (MP) grade of I and the Residual Cancer Burden (RCB) grade of III. The tumor involved the skin and nipple-areolar complex, while the basal and skin surgical margins were negative for carcinoma. Metastatic carcinoma was found in 11 of 18 right axillary lymph nodes. Final pathological stage was ypT4N3M0 (Stage IIIC). Immunohistochemical analysis showed ER (80 % +), PR (80 % +), HER-2 (2+, and FISH -), and Ki-67 (10 % +) (Fig. 2B, C, D). The patient subsequently received radiotherapy and endocrine therapy with tamoxifen (10 mg twice daily, orally). Favorable outcomes were observed during recent follow-up evaluations.

## 3 Discussion

Clinical studies have demonstrated that male patients with breast cancer tend to present with more advanced disease at diagnosis compared to their female counterparts, characterized by larger tumor size and higher rates of axillary lymph node involvement [5]. The present study reports a case of a male patient diagnosed with breast cancer, staged as T4 according to the tumor-node-metastasis (TNM) classification system, and presenting with concurrent multiple lymph node metastases. Gao et al. [6] reported that a case of MBC received neoadjuvant chemotherapy and achieved pathological complete response (pCR). However, the patient in the present study exhibited partial remission, which may be associated with low Ki-67 expression. The occurrence of dual primary malignancies involving MBC and another primary malignant tumor is exceptionally rare, particularly in cases involving leukemia. Previous studies have documented rare instances in which patients, following complete remission of acute lymphoblastic leukemia (ALL), were subsequently diagnosed with MBC several years thereafter [7,8]. Besides, several studies have documented the development of AML in female breast cancer patients following chemotherapy [9,10]. The present study reports the first documented case of a patient with AML-M3a who, following multiple chemotherapy regimens and sustained complete remission, subsequently developed primary MBC. We hypothesize that the series of chemotherapy regimens administered for this patient's AML treatment may have induced somatic gene mutations, potentially leading to the development of MBC. However, comprehensive genomic profiling via next-generation sequencing (NGS) to validate the presence of such mutations was not performed due to prohibitive financial constraints. Consequently, analysis of a potential causal relationship between these two malignancies in this patient was precluded. Although the occurrence of leukemia and primary MBC is rare, it is imperative to prioritize routine clinical breast examinations in male patients with a history of hematologic malignancies to ensure early detection and timely intervention.

## 4 Conclusion

This case report presents the first documented occurrence of MBC in a patient who achieved sustained complete remission of AML, highlighting the need for long-term surveillance of secondary malignancies in AML survivors. The findings may inform clinical decision-making regarding multidisciplinary management strategies for such rare dual malignancies.

## Author contribution

**Miao Deng**: The conception and design of the study. **Weiqiang Qiao**: Data curation, Writing-Original draft preparation. **Peng Li**: Writing-Reviewing and Editing. **Chang Chang**: Supervision, Software. **Mengnan Fan**: Visualization, Investigation.

## Consent

Written informed consent was obtained from the patient for publication of this case report and accompanying images. A copy of the written consent is available for review by the Editor-in-Chief of this journal on request.

## Ethical approval

This study was approved by the Ethics Committee of the First Affiliated Hospital of Henan University of Science and Technology.

## Guarantor

Miao Deng.

## Research registration number

Not applicable.

## Funding

This research did not receive any specific grant from funding agencies in the public, commercial, or not-for-profit sectors.

## Conflict of interest statement

The authors declare that there is no conflict of interest.
